# Early detection of plant virus infection using multispectral imaging and spatial–spectral machine learning

**DOI:** 10.1038/s41598-022-06372-8

**Published:** 2022-02-24

**Authors:** Yao Peng, Mary M. Dallas, José T. Ascencio-Ibáñez, J. Steen Hoyer, James Legg, Linda Hanley-Bowdoin, Bruce Grieve, Hujun Yin

**Affiliations:** 1 Department of Electrical and Electronic Engineering, University of Manchester, Manchester, UK; 2 Department of Plant and Microbial Biology, North Carolina State University, Raleigh, NC USA; 3 Department of Molecular and Structural Biochemistry, North Carolina State University, Raleigh, NC USA; 4 Department of Ecology, Evolution, and Natural Resources, Rutgers University, New Brunswick, NJ USA; 5 International Institute of Tropical Agriculture (IITA), Dar es Salaam, Tanzania

**Keywords:** Electrical and electronic engineering, Virulence

## Abstract

Cassava brown streak disease (CBSD) is an emerging viral disease that can greatly reduce cassava productivity, while causing only mild aerial symptoms that develop late in infection. Early detection of CBSD enables better crop management and intervention. Current techniques require laboratory equipment and are labour intensive and often inaccurate. We have developed a handheld active multispectral imaging (A-MSI) device combined with machine learning for early detection of CBSD in real-time. The principal benefits of A-MSI over passive MSI and conventional camera systems are improved spectral signal-to-noise ratio and temporal repeatability. Information fusion techniques further combine spectral and spatial information to reliably identify features that distinguish healthy cassava from plants with CBSD as early as 28 days post inoculation on a susceptible and a tolerant cultivar. Application of the device has the potential to increase farmers’ access to healthy planting materials and reduce losses due to CBSD in Africa. It can also be adapted for sensing other biotic and abiotic stresses in real-world situations where plants are exposed to multiple pest, pathogen and environmental stresses.

## Introduction

The advent of digital technology has been making an impact on growing number of areas including agriculture. There is a pressing need for better management of limited resources and optimisation of cultivation practice, including early detection of plant diseases. While end-point PCR is often the preferred diagnostic method for detection of viral nucleic acid in field-collected samples, it is dependent on expensive instrumentation, time consuming and often cannot reliably detect virus early in infection, as seen for cassava brown streak disease (CBSD)^1^. Imaging technology has been applied to the analysis of plant conditions and nutrition levels, either based on visual traits or certain spectral properties reflected by the conditions or diseases^2^. Hyperspectral imaging (HSI) and multispectral imaging (MSI) have become increasingly available and affordable techniques that offer many advantages over conventional RGB imaging. RGB imaging has been used to recognise visual symptoms of the disease^3^, while plant nutritional conditions and metabolic or biotic changes due to disease may be reflected in certain spectral wavelengths beyond the RGB channels^3–5^. These subtle signs in vast amounts of spectral and spatial imaging data can be successfully detected using advanced machine learning techniques. With the rapid advancement of imaging sensors, MSI systems have become smaller and are able to be applied in real-time and in-field^6^. This paper describes the application of a custom built active MSI (A-MSI) device and a machine learning method that leverages both spectral and spatial information of the imagery data for early detection of CBSD.

Cassava, *Manihot esculenta* Crantz, produces starchy tuberous roots and is one of the important staple food crops in the developing world^7,8^. It is cultivated primarily by smallholder farmers. Cassava production in Africa is limited by two viral diseases, cassava mosaic disease (CMD) and CBSD. Together these diseases cause severe economic losses and threaten food security^9^. CMD has been extensively studied and sources of endogenous resistance have been identified and deployed^10–16^. Unfortunately, many farmer-preferred cassava cultivars, like the CMD2-resistant cultivar TME204, are highly susceptible to CBSD. CBSD, which was first reported in the coastal areas of Tanzania^17^, has emerged recently as serious threat to food production^14,18^. The rapid spread of CBSD throughout east and central Africa resulted in research targeting development of new cassava cultivars that are resistant to both CBSD and CMD^8,19–21^.

CBSD is caused by two closely related RNA viruses, cassava brown steak virus (CBSV) and Ugandan cassava brown streak virus (UCBSV), in the *Ipomovirus* genus of the *Potyviridae* family. These viruses are transmitted from plant to plant by the whitefly *Bemisia tabaci*^22^. The viruses are also propagated from one season’s crop to the next through the use of stem cuttings obtained from infected plants. CBSD typically induces only mild foliar symptoms that can be difficult to discern. The subtle symptoms can make it difficult even for experts to identify infected plants in the field, and the call is often complex and based on many visual cues. However, the tuberous roots of infected cassava plants have prominent necrotic lesions that can spread throughout the entire root structure, rendering it inedible. Because the necrotic lesions or root rot are only discovered when cassava is harvested, farmers often do not know that their crop is infected with CBSD until harvest at 9–12 months after planting.

Although considerable effort has been devoted to the search for strong sources of resistance to CBSD, the progress has been slow because of the lack of a rapid, reliable method for diagnosing CBSD during early stages of infection. Historically, diagnosis is based on subtle symptoms^23^ and scoring requires visible symptoms characteristic of established infections and does not distinguish resistant from tolerant plants. The use of molecular techniques to screen for the presence or absence of viral RNA has only recently been implemented in a few African national research programmes because of the technical requirements, cost, and time constraints involved in screening large numbers of plants^8,21,24^. To address these constraints, we have developed an active multispectral imaging (A-MSI) sensor system enhanced with machine learning as a screening platform for virus infection.

Multispectral imaging of plant viral infections generates massive amount of data. Machine learning techniques have become the most efficient means of extracting useful information from the wealth of the data available and for detecting the underlying relationships between certain mechanisms or functions under study and a large number of contributing parameters. Our previous studies^25,26^ have used feature selections and a novelty detection classifier to distinguish healthy sugar beet plants from rust-diseased plants or stressed from control Arabidopsis plants using laboratory-based hyperspectral imaging systems. Performance was significantly higher than conventional vegetation indices such as NDVI (the normalized difference vegetation index).

In the study reported here, an A-MSI device and machine learning were combined to probe the early detection of CBSD. Such an approach alleviates the burden of using an expensive and precise MSI system. Machine learning techniques can effectively make sense of plant conditions even with a low-cost, compact and less precise MSI device. Combining spectral and spatial features of the scans, machine learning identified significant differences at a high confidence between healthy cassava plants and plants inoculated with UCBSV in four experimental trials. The approach reliably detects CBSD much earlier and in a faster and much less invasive manner than end-point PCR. The integrated handheld device with advanced machine learning should make it possible to detect disease in cassava fields early in the growing season, at a time when farmers can replant with virus-free cassava cuttings and should improve the efficiency of the work of cassava breeders in selecting for resistance to CBSD.

**Table 1:** Experimental design of the Cassava-TME204-UCBSV A-MSI trials.

Trial	Cultivar	Susceptibility		Groups	Treatment	No. of plant	Leaf position
		CMD	UCBSV				
1	TME204	Resistant	Susceptible	Control	Not inoculated	24	Leaf 2 for 7 dpi, leaf 3 for 28 and 53 dpi, and leaves 2 and 6 for 88 dpi
				Mock	Empty injection	12	
				Infected	Inoculated by UCBSV	12	
2				Control	Not inoculated	18	Leaf 2 for 14, 28 and 54 dpi
				Mock	Empty injection	18	
				Infected	Inoculated by UCBSV	18	
3				Control	Not inoculated	18	Leaf 2 for 7, 14, 21, 28, 52 and 59 dpi
				Mock	Empty injection	18	
				Infected	Inoculated by UCBSV	18	

**Figure 1:**
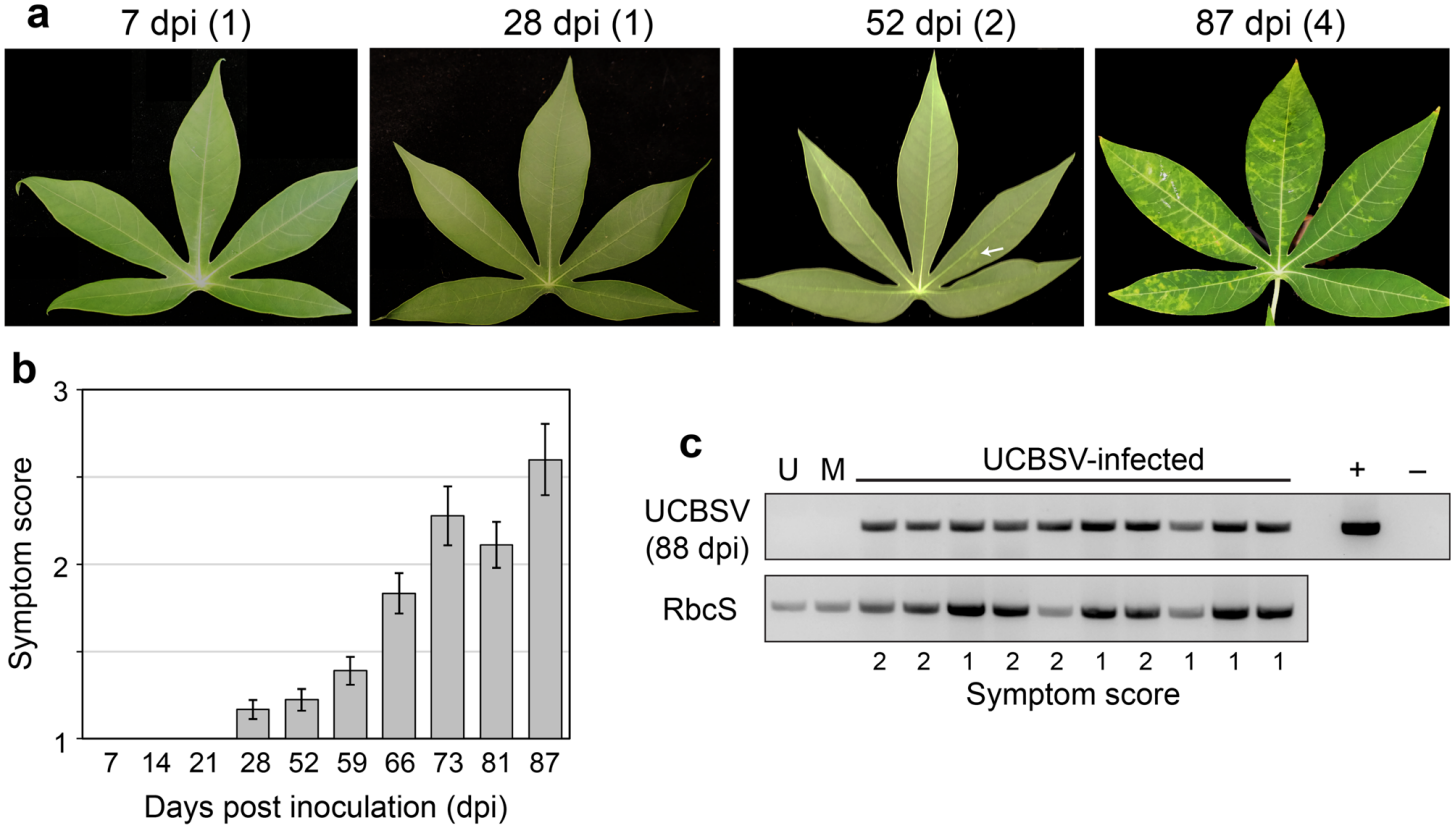
Infection results for Trial 2. (**a**) Images of leaves from TME204 plants at 7, 28, 52 and 87 dpi. The symptom score of each leaf is in parentheses. The white arrow points to faint yellow blotches characteristic of a symptom score of 2. (**b**) Average symptom scores from 7 to 87 days. (**c**) End-point RT-PCR of total RNA extracts isolated from untreated (U), mock-inoculated (M), and UCBSV-inoculated plants at 88 dpi. The upper panel shows a 445-bp band corresponding to UCBSV. The lower panel shows a 619-bp band corresponding to a cassava RbcS transcript, which served as a positive control for the isolation of amplifiable RNA.

## Results

### Experimental settings

In three independent trials, we used the cassava cultivar TME204, which is susceptible to CBSD, to generate three treatment groups—control, infected and mock-inoculated (18 plants in each group, except for Trial 1—see Table 1). The infected group was inoculated with plasmid DNA corresponding to an infectious clone for the UCBSV Kenyan isolate 125^27–29^. The mock group was inoculated with an ‘empty’ control *E. coli* plasmid using the same protocol and the untreated group was not subjected to inoculation. All of the plants were grown together in an insect-free plant growth chamber for the duration of each experiment. The three treatment groups were visually indistinguishable at 7, 14 and 21 days post inoculation (dpi) (Fig. 1, panel B). At 28 dpi, a few plants (15%) inoculated with UCBSV displayed very mild symptoms on a single leaf. For plants showing symptoms, their severity slowly increased over the next 8 weeks. By 87 dpi, all of the plants in the UCBSV-inoculated treatment group displayed symptoms with an average symptom score of 2.6 (out of 4), highlighting the mild nature of the symptom phenotype (Fig. 1, panel A). UCBSV infection was confirmed in 10 plants at 88 dpi by end-point RT-PCR of viral RNA (Fig. 1, panel C). We did not analyze viral RNA in the other 8 UCBSV-inoculated plants because the RcbS positive control could not be amplified from the samples. Viral RNA could also be detected at 52 dpi in some plants but with variable results. During the timeframe of the experiment, no symptoms were seen on plants in the other treatment groups and the RT-PCR results were negative for the control and mock groups. These results indicate that inoculation was highly efficient, consistent with the original report of this infectious clone^29^, but do not guarantee that 100% of plants in the virus-inoculated group were infected. Some of the classification results presented below were therefore done using only the 10 plants with PCR-confirmed infection (“Trial 2 with PCR”).

**Figure 2:**
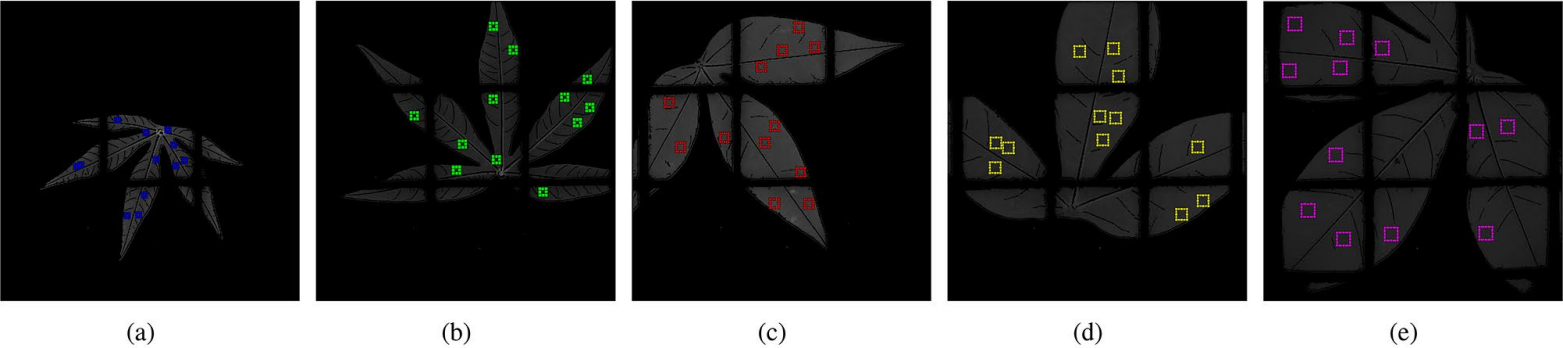
Randomly selected patches of various sizes sampled from leaf areas, avoiding leaf clapping grid and main veins. Patch sizes (pixels): (**a**) $16 \times 16$, (**b**) $24 \times 24$, (**c**) $32 \times 32$, (**d**) $40 \times 40$ and (**e**) $48 \times 48$.

### Scanning of cassava leaves

A single leaf (usually the second visible leaf from the apex) from each plant was screened using the A-MSI device, producing leaf images across 14 wavelengths. Twelve small patches were automatically cropped out from random spatial locations in the leaf area of each image at each wavelength, avoiding the leaf clapping grids and main leaf veins. This represents a simple approach to considering spatial variation in contrast to using the whole leaf for classification. Cropped patches were of various sizes, varying from 16 × 16 to 48 × 48 pixels, which covered sufficient spatial variations. Examples are shown in Fig. 2.

### Performance for cassava disease detection

From each cropped patch region of a scanned leaf across the wavelengths, a variety of spectral and spatial features were extracted and investigated, including six vegetation indices (VIs), patch-based spectra, as well as patch-based texture extracted by Markov random field (MRF) probabilistic texture model. For examples, patch-based spectra are the averaged spectral reflectance values across all wavelengths within a patch region, while patch-based MRF textural features are the MRF model coefficients. Details of various vegetation indices are given in the “Methods” section. Support vector machine (SVM) was used as the classifier, along with various information fusion schemes, including the proposed probabilistic decision fusion method (ProbDecFus) for more reliable classification. For example, spatial–spectral fusion combines spatial features (MRF) and spectral features (e.g. patch-based spectra) for classification or integrates classifiers built on spatial features and classifiers built on spectral features. Details of various schemes are described in the “Methods” section.

**Table 2:** Classification accuracy (%) at 28 dpi on leaf scans of three trials of Cassava-TME204-UCBSV and one trial of Cassava-Kiroba-UCBSV.

Groups	Methods	TME204				Kiroba
		Trial 1	Trial 2	Trial 2 w PCR	Trial 3	
Control versus infected	Vegetation indices	56.0 ± 13.1	87.7 ± 10.0	89.4 ± 9.3	73.9 ± 12.0	69.72 ± 21.22
	Spectral reflectance (whole leaf)	67.6 ± 25.0	97.6 ± 8.3	96.9 ± 9.8	88.8 ± 16.4	87.41 ± 18.43
	Spectral reflectance (patch-based)	78.9 ± 13.7	95.8 ± 5.8	96.1 ± 5.0	90.8 ± 11.3	87.98 ± 12.49
	Spatia–spectral fusion (patch-based)	67.7 ± 13.6	94.1 ± 7.3	95.9 ± 6.3	89.1 ± 11.9	82.58 ± 14.50
	Decision fusion (average)	76.9 ± 13.8	95.1 ± 6.6	95.6 ± 6.1	90.3 ± 11.5	83.67 ± 16.38
	Spectral reflectance (patch-based voting)	87.2 ± 17.5	98.7 ± 5.6	98.2 ± 6.4	93.5 ± 14.5	90.60 ± 15.74
	Spatial–spectral fusion (patch-based voting)	78.6 ± 20.9	98.3 ± 6.3	98.0 ± 5.8	91.7 ± 15.7	85.19 ± 20.07
	ProbDecFus (patch-based)	79.0 ± 13.6	95.7 ± 6.3	96.5 ± 5.8	90.8 ± 11.4	88.12 ± 12.83
	ProbDecFus (patch-based voting)	87.3 ± 17.4	98.5 ± 6.9	98.6 ± 5.1	93.6 ± 14.8	91.10 ± 15.74
Mock versus infected	Vegetation indices	53.6 ± 16.2	57.0 ± 14.9	56.3 ± 14.8	56.6 ± 17.5	63.21 ± 23.57
	Spectral reflectance (whole leaf)	52.1 ± 21.8	75.3 ± 26.3	67.3 ± 22.3	76.1 ± 27.0	72.35 ± 28.27
	Spectral reflectance (patch-based)	65.7 ± 13.3	72.6 ± 19.2	67.7 ± 17.6	76.9 ± 18.4	66.45 ± 22.80
	Spatial–spectral fusion (patch-based)	52.8 ± 13.3	71.9 ± 17.3	66.9 ± 17.7	74.7 ± 16.5	69.66 ± 22.29
	Decision fusion (average)	63.8 ± 15.0	70.9 ± 17.9	66.0 ± 16.9	74.2 ± 19.9	67.70 ± 24.84
	Spectral reflectance (patch-based voting)	75.5 ± 20.8	75.4 ± 24.7	70.9 ± 24.1	81.0 ± 22.8	69.42 ± 27.54
	Spatia–spectral fusion (patch-based voting)	57.1 ± 25.8	78.4 ± 25.6	71.1 ± 24.6	81.3 ± 21.8	76.37 ± 27.36
	ProbDecFus (patch-based)	66.0 ± 13.3	72.3 ± 18.7	67.3 ± 17.4	77.6 ± 18.2	66.97 ± 23.30
	ProbDecFus (patch-based voting)	75.9 ± 20.8	75.1 ± 24.5	70.2 ± 23.7	81.8 ± 22.6	69.47 ± 27.72
Control versus mock	Vegetation indices	55.3 ± 16.9	74.6 ± 15.5	N/A	67.8 ± 14.7	50.89 ± 16.45
	Spectral reflectance (whole leaf)	60.8 ± 27.7	88.6 ± 18.9		79.4 ± 25.6	90.54 ± 15.36
	Spectral reflectance (patch-based)	63.6 ± 17.7	91.4 ± 9.9		76.3 ± 15.5	84.56 ± 14.1
	Spatial–spectral fusion (patch-based)	59.8 ± 15.5	90.4 ± 11.0		77.1 ± 15.2	81.80 ± 13.29
	Decision fusion (average)	62.0 ± 17.9	88.5 ± 12.3		75.9 ± 15.3	80.11 ± 15.17
	Spectral reflectance (patch-based voting)	70.7 ± 25.5	93.4 ± 11.5		84.3 ± 20.2	88.12 ± 19.01
	Spatial–spectral fusion (patch-based voting)	66.9 ± 26.2	93.3 ± 14.0		84.7 ± 19.7	87.10 ± 20.52
	ProbDecFus (patch-based)	63.2 ± 17.7	91.4 ± 10.4		76.5 ± 15.4	84.95 ± 14.22
	ProbDecFus (patch-based voting)	79.0 ± 25.6	93.1 ± 11.9		84.5 ± 19.9	88.19 ± 19.23

‘Trial 2 w PCR’ of Cassava-TME204-UCBSV denotes the classification results of the models re-trained only on those leaves that were later confirmed of infection with PCR at 88 dpi.

Performance for detecting CBSD in TME204 has been investigated with various methods described in the “Methods” section for utilising these spectral and spatial features as well as classifier fusions, divided into to the following three categories, with corresponding results at 28 dpi shown in Table 2. For each of the three groups or pairs (control vs. infected, mock vs. infected, and control vs. mock), results are divided into the following three subgroups.

1. Conventional spectral methods (1st two rows in the table, i.e. Vegetation indices and Spectral reflectance (whole leaf)). In the vegetation indices method, each of six VIs was calculated and averaged from cropped patch regions and six VIs were then concatenated for classification. The spatial reflectance (whole leaf) method refers to using the averaged spectral reflectance (spectrum) from each leaf.
2. Spatial–spectral methods (next two rows). Spectral reflectance (patch-based) refers to using patch-based spectra for classification. Instead of using averaged spectra from entire leaves, patch-based spectra represents the simplest spatial–spectral approach. The spatial–spectral fusion (patch-based) method refers to the use of concatenated patch spectrum and MRF texture parameters for classification.
3. Spatial–spectral and classifier fusion methods (the remaining five rows). Decision fusion (average) refers to fusion of three classifiers trained on VIs, spectral reflectance and MRF texture parameters extracted from patches. The patch-based voting scheme regards a leaf as infected when at least half of the patches extracted from the leaf are classified as infected. The scheme was used with either spectral information (spectral reflectance (patch-based voting)) or spatial–spectral information, i.e. concatenated patch spectrum and MRF parameters (spatial–spectral fusion (patch-based voting)). The proposed probabilistic decision fusion method (ProbDecFus) combines VIs, MRF texture features and spectral reflectances to generate reliable classification. When the method is used on patches, we refer to it as ProbDecFus (patch-based). When it is used with patch-based voting, the method becomes ProbDecFus (patch-based voting).

For a fair and comprehensive comparison, we separately trained the classifiers on three pairs of sample sets: control versus infected, mock versu infected, and control versus mock. A leave-one-leaf-out scheme was adopted in Trial 2 and Trial 3 of TME204 as the numbers of leaf samples in the three conditions (control, infected and mock) were 18:18:18. Trial 1 of TME204 however had unbalanced sample numbers (24:12:12). We therefore randomly selected half of the control leaves each time and then performed the leave-one-leaf-out training. The random selection process of control samples was repeated at least 5000 times and the averaged classification results were produced. For finding the best hyperparameters of the SVM in each training, we randomly chose half of the training samples as the validation set and optimised the SVM classifier using the grid search algorithm.

As shown in Table 2, at 28 dpi the proposed ProbDecFus with patch-based voting achieved a classification accuracy of 87.3% for Trial 1, 98.5% for Trial 2 and 93.6% for Trial 3 on control versus infected. The methods using patch-based spatial information (fusion or not) greatly boosted classification compared to using the whole leaf. Using additional MRF texture features further improves and stablises the performance. For the “Trial 2 w PCR” results, the classifiers were trained only from those leaves with detected UCBSV by end-point RT-PCR (Fig. 1, panel C). The similarities between this column and “Trial 2” column confirm the effectiveness of the A-MSI device and classification method in detecting CBSV at 28 dpi.

**Figure 3:**
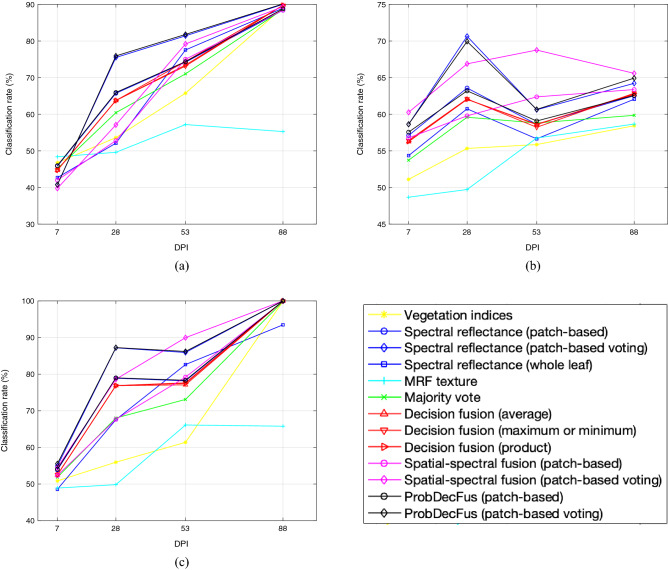
Classification accuracy (%) from leaf scans on Cassava-TME204-UCBSV Trial-1, at 7, 28, 53 and 88 dpi, respectively, (**a**) control versus infected, (**b**) mock versus infected, and (**c**) control versus mock.

The progressive detection performances on Trial 1 are shown as an example as it has the longest time course. Figure 3 depicts the classification results of various methods over leaf samples at 7, 28, 53 and 88 dpi. These graphs illustrate again that combining spatial and spectral information gives an edge over other ways of utilising the available information and significantly outperforms the use of vegetation indices. It is worth noting that although MRF is a powerful model for describing spatial dependence, it does not deliver convincing results when used alone.

### Performance on a tolerant cultivar

**Figure 4:**
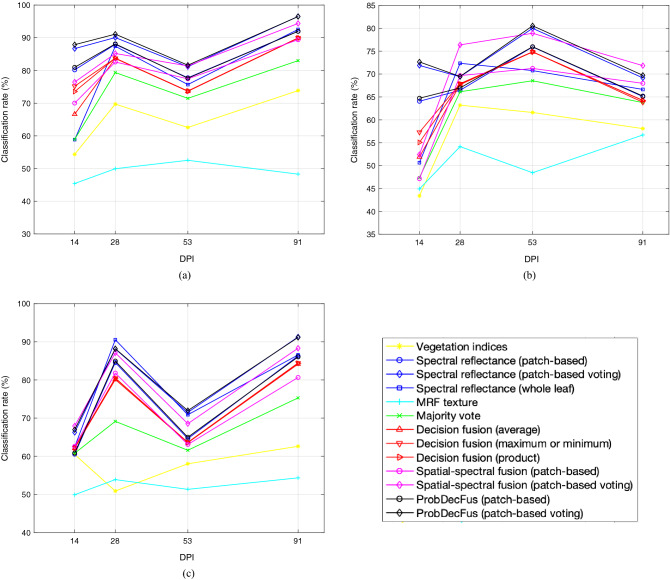
Classification accuracy (%) from leaf scans on Cassava-Kiroba-UCBSV dataset, at 14, 28, 53 and 91 dpi, respectively, (**a**) control versus infected, (**b**) mock versus infected, and (c) control versus mock.

The CBSD tolerant cultivar, Kiroba^30^, was also tested in a single trial to verify early detection of CBSD. Eighteen plants in each group (control, infected and mock) were used in the trial using an experimental procedure similar to that for TME204 as described before. Scans of the leaves took place at 14, 26, 53 and 91 dpi. No clearly visible symptoms were observed during the course of the trial. Classifications between control versus infected, mock versus infected and control vs. mock were performed and results of 28 dpi are shown in the “Kiroba” column of Table 2. Performances across all time courses are plotted in Fig. 4. As can be seen these results are in line with that of TME204, indicating early CBSD detection at 28 dpi in a tolerant, asymptomatic cultivar. While at 14 dpi, most classifiers, except for Vegetation Indices, whole leaf and MRF, can already separate with over 70% accuracy between the infected and control. After 28 dpi, classification performances do not seem to increase further, unlike in the case of TME204; this could be well due to the tolerant nature of Kiroba and its restrictive virus accumulation^30^.

## Discussion

Several technologies have been used for diagnosis of plant viral diseases, including, enzyme-linked immunosorbent assay (ELISA), loop mediated isothermal amplification (LAMP)^31^, and PCR. All three have been used to detect CBSV and UCBSV, with PCR being the most widely used. RNA isolation from non-model plants like cassava is often technically challenging, whereas the scanning approach described here does not require RNA isolation. To date, the high cost and need for laboratory resources have been prohibitive for mass deployment of these technologies for real-time, in-field detection of CBSD. Imaging offers an alternative to indirectly sense subtle biological changes reflected in plant leaves as a result of viral infection. Such an approach is almost instant once configured and can be portable, hence offering the potential for widespread, low-cost deployment. The results in this study also show that such changes can be detected at a very early stage, well before symptoms emerge, whereas end-point PCR depends on viral RNA accumulation to detectable levels, which occurs later in infection. Across the three trials of TME204, the available common sampling points were at 7, 28 and 53 dpi, as shown in Table 1. Trials 1 and 3 were also scanned at 14 dpi and detection of differences was also highly probable (> 80%). Further optimization of the spectral profile of the A-MSI device may enable still earlier detection of CBSD. In principle, early detection of infection in primary whitefly-infected leaves could even enable removal of infected tissue before the virus can move systemically thus eliminating infection to preserve the yield of individual plants.

Machine learning approaches for analysis of MSI data can effectively even out inaccuracies of the imaging to some extent. Even with intuitively chosen wavelengths, as compared to those strictly optimized through many rounds of cross-validation process, machine learning has proven useful and effective. This in principle is in line with its broad deviation from the traditional orthogonal approach to information processing. With an active MSI system, more crop or growth conditions could be investigated using the modulation properties of various wavelengths of electromagnetic spectrum in response to metabolic changes in the organism, as well as translucent properties of the plant leaves, extending the imaging approach from spectral and spatial to temporal and transitive. We are optimistic that refinements of this approach in future field trials may be useful for early detection of infection in a wide range of crop pathosystems.

To further demonstrate the generalizability of the developed spatial–spectral machine learning method to other HSI/MSI applications, a public benchmark HSI dataset was used. The Indian Pine dataset is a HSI image dataset on land coverage^32^. Each image is of 145 by 145 pixels with a spatial resolution of 20 m covering 16 different crops, provided in the ground truth reference as detailed in^32^. The dataset was captured by the AVIRIS sensor at the Indian Pines test site in June 1992. The data contains a subset of a full scene that covers portions of Northwestern Tippecanoe County, IN, USA. The dataset is widely used in HSI analysis for validating classification efficiency. Comparisons with the state-of-the-art methods are presented in Table 3. The classification was performed using a 5-fold cross-validation strategy, and repeated 10 times to achieve satisfactory precision. Convolutional neural networks (CNNs) were used on the extracted spectral-spatial features. The baseline model, named 2D-CNN, consisted of seven main blocks (architecture: 1 × 20-(8C3-8C3)-16R3-32R3-64R3-128R3-256R3-(512FC-16FC)). The first block used two convolutional layers, each containing 8 filters of 1 × 3 with zero padding. After subsequent five residual blocks, two fully connected layers were used with a softmax layer to produce probabilistic output over each class. Batch normalisation was inserted in each convolutional layer between the convolutional and ReLU activation. A max-pooling layer was also inserted in each residual block after the addition function. The developed spatial–spectral Net ($\hbox {SSFNet}_{\text{2D}}$) had the same architecture as the baseline except that the input was further combined with MRF texture parameters extracted from the 25 × 25 surrounding neighbourhood of the centre pixels. The networks were trained using the Adam optimiser for 200 epochs with a batch size of 4. The learning rate started at 0.001 and was decreased using the polynomial scheduler. Again, the inclusion of spatial features was beneficial, resulting in more accurate and more stable classification results.

**Table 3:** Class-specific accuracies (%) on Indian Pines dataset.

Class	3D-CNN-LR^33^	RNN-GRU-PReTanh^34^	Feature-ensemble ND-SVM^25^	CNN-MRF^35^	HSINet^36^	$\hbox {U}_{\text {Hfe}}$ $\hbox {SRVAE}_{11}$^37^	2D-CNN	$\hbox {SSFNet}_{\text{2D}}$
1	100	70.6	99.9 ± 0.1	86.5	100	89.6	90.7 ± 7.5	95.3 ± 4.3
2	96.3 ± 1.1	70.3	66.4 ± 1.4	91.5	66.9	89.4	97.6 ± 1.3	98.4 ± 1.0
3	99.5 ± 0.7	81.5	82.8 ± 1.0	96.4	62.4	85.1	97.8 ± 1.6	98.7 ± 1.2
4	100	90.2	89.9 ± 1.2	96.2	100	82.0	97.8 ± 2.4	99.0 ± 1.8
5	99.9 ± 0.2	92.0	94.6 ± 0.6	99.5	83.2	92.6	96.4 ± 2.5	97.8 ± 2.4
6	99.8 ± 0.3	96.1	99.3 ± 0.1	99.8	98.0	96.7	98.6 ± 1.3	99.3 ± 0.8
7	100	84.8	99.9 ± 0.1	78.0	100	34.8	84.6 ± 17.6	93.0 ± 9.6
8	100	59.6	99.6 ± 0.1	98.8	99.7	98.6	99.9 ± 0.3	99.9 ± 0.1
9	100	86.2	99.9 ± 0.1	100	100	93.8	84.9 ± 17.5	93.9 ± 9.8
10	98.7 ± 1.0	99.4	92.2 ± 0.7	94.3	77.5	89.9	97.1 ± 1.9	98.4 ± 1.4
11	95.5 ± 1.2	85.0	77.7 ± 1.0	96.5	78.4	93.2	99.0 ± 0.6	99.4 ± 0.5
12	99.5 ± 0.4	77.6	83.2 ± 1.2	91.9	75.0	85.5	96.8 ± 2.0	97.6 ± 2.7
13	100	95.6	99.8 ± 0.1	98.9	99.5	99.0	98.7 ± 2.2	99.1 ± 1.6
14	99.6 ± 0.6	84.6	95.7 ± 0.2	98.4	96.5	96.7	99.7 ± 0.5	99.9 ± 0.2
15	99.5 ± 1.3	90.9	86.2 ± 1.1	91.5	69.1	80.1	98.9 ± 1.4	99.2 ± 1.3
16	99.3 ± 1.08	100	99.9 ± 1.0	97.9	100	92.9	87.8 ± 7.5	95.3 ± 5.9
OA	97.6 ± 0.4	88.6	–	96.1	83.0 ± 0.2	91.4	98.1 ± 0.4	98.9 ± 0.4
AA	99.2 ± 0.2	85.3	91.7 ± 0.1	94.8	87.9 ± 0.2	87.5	95.4 ± 3.1	97.8 ± 2.8
$\kappa \times 100$	97.0 ± 0.5	73.7	–	95.8	81.9 ± 0.2	90.2	–	–

## Methods

### Active multispectral imaging (A-MSI) system

**Figure 5:**
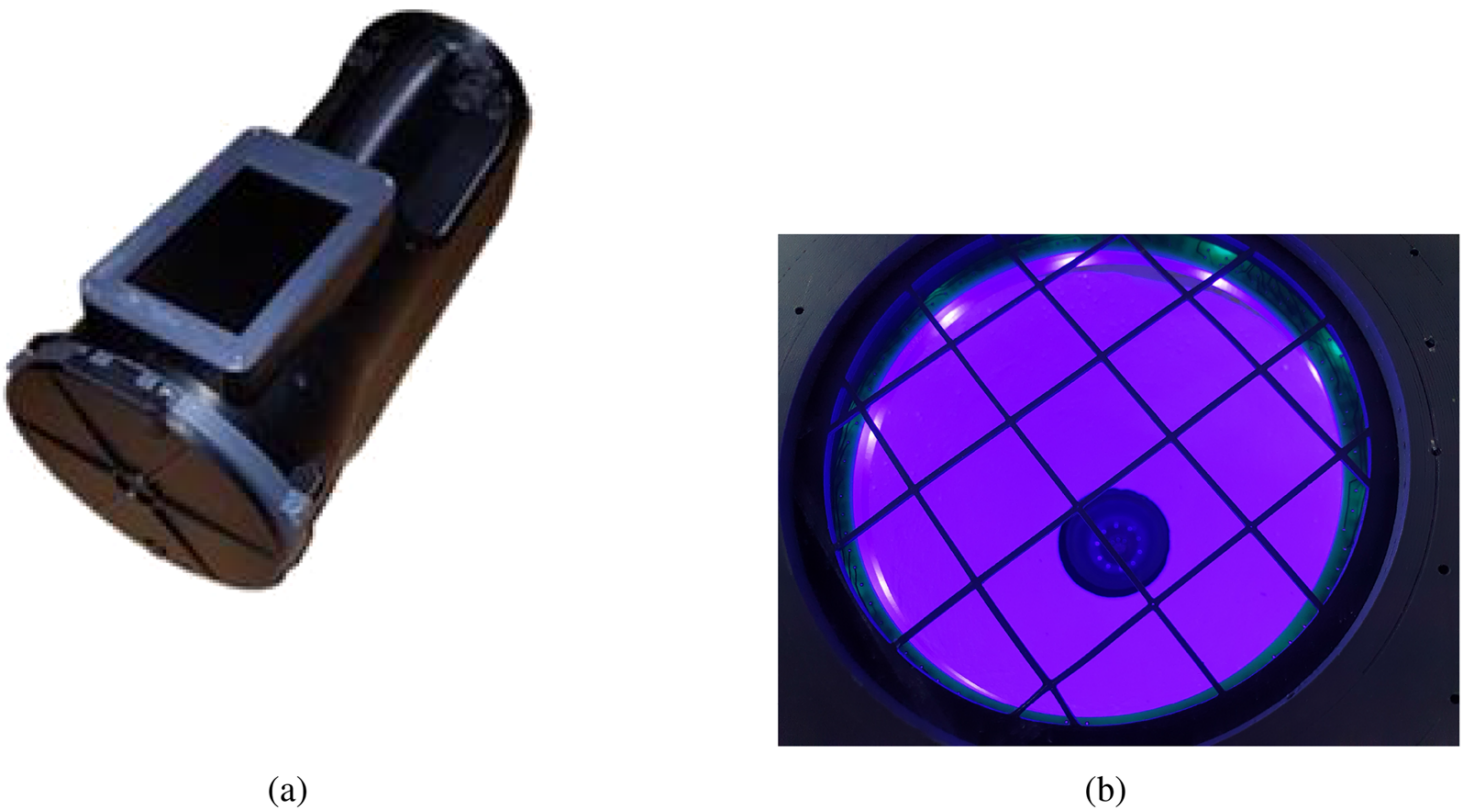
(**a**) Photo of the developed A-MSI system and (**b**) its LED ring and sensing chamber.

A handheld active multispectral imaging (A-MSI) prototype, developed at the e-Agri Sensors Centre, the University of Manchester, was used to obtain the data presented in this study. The sensor system exploits a modified proprietary digital imaging detectors appropriately engineered within the active optical assembly, also to enable the vastly overlapping ‘Nth-order’ molecular vibrational harmonics, from the near-infrared and visible 2D time-series data, to be deconvoluted. Isotropic illumination is achieved, with minimised specular reflectance, via a combination of an integrating hemisphere, optical diffuser and appropriately arranged narrow-band semiconductor sources (LEDs). The latter cover 15 wavebands using 10 LEDs per waveband, at the wavelengths detailed in the Table 4. Custom drive electronics are then used to enable the multispectral frames to be compiled within a parallel processing unit (NVDIA Jetson Nano). The variant of A-MSI adopted in the study is distinct from more traditional passive multispectral imaging (MSI) systems^38^ as closed-loop control of the illumination power at each detection band enables highly repeatable measurements to be undertaken with significantly greater signal-to-noise ratio (SNR) than that by a filtered or dispersive-element based passive MSI sensor-system. This is due, in part, to the variability in illumination angle, spectral composition and polarisation of ambient illumination. The prototype instrument used in the study is shown in Fig. 5, which depicts the as-built unit and the inset measurement chamber, relative position of the LED array, and camera assembly within the integrating hemisphere. The system automatically calibrates the illumination level for each band at the start of each scanning process.

**Table 4:** Detailed wavelength information used in the A-MSI system.

LED No.	Band no.	Wavelength (centre band)
1	8	395 nm
2	9	415 nm
3	10	470 nm
4	11	528 nm
5	12	532 nm
6	13	550 nm
7	14	570 nm
8	0	585 nm
9	1	590 nm
10	2	610 nm
11	3	625 nm
12	4	640 nm
13	5	660 nm
14	6	700 nm
15	7	880 nm
Null	15	No LED

### Cassava cultivation and virus inoculation

Cassava cuttings (*Manihot esculenta* cultivar TME204 or Kiroba) were originally provided by J. Ndunguru (Tanzania Agricultural Research Institute). Import and containment of plant cuttings and the UCBSV infectious clone (pLX-UCBSVi) followed international, U.S. (Department of Agriculture Animal and Plant Health Inspection Service), and institutional guidelines. Plants were propagated at 28 °C in a 12-h light/12-h dark cycle. For each experiment, 18 plants at the 6-leaf stage were inoculated under the apical meristem area using a microsprayer and 40 psi helium to deliver gold particles coated with plasmid DNA^39^ (Venganza, Inc.). Each plant was inoculated with 1.67 μg of plasmid DNA corresponding to pLX-UCBSVi (infected)^28,29^ or pUC119 (mock). pLX-UCBSVi contained an E35S expression cassette driving transcription of UCBSV Ke_125 (GenBank accession KY825166^27^). Untreated plants (18) were not subjected to the inoculation treatment. Plants were monitored for symptoms and were scored on a scale of 1 (no symptoms), 2 (small yellow blotches on 1 leaf), 3 (yellow blotches on two leaves), and 4 (yellow blotches and yellowing along veins on multiple leaves).

TME204 samples (1 mg) were collected at 88 dpi near the petiole of leaf 2 (relative to the plant apex), flash-frozen in liquid nitrogen, and stored at − 18 °C. Leaf samples were ground in a Retsch Mixer Mill, followed by RNA extraction using a Qiagen RNeasy Plant Mini kit. RNA concentration was measured using the Qubit RNA BR Assay Kit (Invitrogen). cDNA was synthesized in reactions containing 0.5 μg total RNA, oligo d(T)18 mRNA primer (60 μM), RNasin (40 U/μL), dNTPs (10 mM), and M-MuLV reverse transcriptase (200 U/μL) using the following conditions: 5 min at 25 μC , 60 min at 42 μC, 20 min at 65 μC. cDNA (5 μL) was amplified in PCR reactions comprising 10 pMol primers, 10 mM dNTPs, and Hot Start Taq polymerase (NEBioLabs). UCSBV RNA was amplified across the 3′ NIa-Pro and 5′ NIb coding sequences using the primer pair—UCBSV-F (GGGTTCCATAGTGGTGTGATTAG) and UCBSV-F (CTCGAACTGGCTCATTGTACTT). The cassava *RbcS* transcript (Manes.05G137400.1), which served as a positive control for mRNA and cDNA quality, was amplified using the primer pair—RBC-1 (CTACTATGGTGGCTCCGTTC) and RBC-2 (CCGTTCAGTCGGAGAAACTC). Both sequences were amplified for 30 cycles using the following conditions: 95 °C denaturation for 60 s, 51 °C annealing for 60 s, 68 °C extension for 60 s. The PCR products were resolved on 1% agarose gels. The UCBSV products were purified using the Qiagen PCR purification kit and verified by Sanger sequencing.

### Cassava leaf MSI acquisition

Leaves were detached from the TME204 plants at 7, 28, 53 and 88 dpi and from Kiroba plants at 14, 26, 53 and 91 dpi. The adaxial and abaxial surfaces of each leaf were scanned using a handheld multispectral imaging instrument. The leaves were sampled from the same position on each plant (leaf 2 or leaf 6 counting from the plant apex). The plants were also scored for symptom development at the same dpi using a scale from 1 (no visible symptoms) to 4 (severe symptoms). Details of the experimental design are shown in Table 1.

### Data preprocessing

In the experiments, multispectral scans of cassava leaves were sampled by automatically cropping out patches. Twelve patches were cropped out at random spatial locations of the leaf region from each leaf scan. For spectral analysis, the number of pixels from the cropped leaf area were averaged over each wavelength range to reduce the variability in pixel intensities and produce the spectral signature. Examples are shown in Fig. 2. Such patch-based spectral information represents the simplest approach to consider spatial variation. Averaged reflectance across the entire leaf was used for comparisons, in which leaf segmentation was performed and average reflectance calculated.

### Vegetation indices (VIs) calculation

The spectral signatures from each cropped patch were extracted and averaged to calculate empirical VIs. Based on the 14 wavelength bands provided by the A-MSI, six empirical indices were extracted and analysed to study plant properties and conditions. The primary formulation is the Carter index (CI), a strong indicator for plant stress, which measures the ratio between reflectance at 695 nm and 420 nm^40^,

1 $$\begin{aligned} \text {CI}=\frac{R_{695}}{R_{420}}. \end{aligned}$$

The modified chlorophyll absorption in reflectance index (MCARI) measures the depth of chlorophyll absorption at 670 nm relative to the green reflectance peak at 550 nm and the reflectance 700 nm^41^,

2 $$\begin{aligned} \text {MCARI}=[(R_{700}-R_{670}-0.2(R_{700}-R_{550})]\frac{R_{700}}{R_{670}}. \end{aligned}$$

The optimised index transformed chlorophyll absorption in reflectance index (TCARI) was studied as being more sensitive to chlorophyll content, thus avoiding the influence by canopy and soil reflectance values^42^,

3 $$\begin{aligned} \text {TCARI}=3\left[\left(R_{700}-R_{670}-0.2(R_{700}-R_{550}\right)\left(\frac{R_{700}}{R_{670}}\right)\right]. \end{aligned}$$

The photochemical reflectance index (PRI) measures the normalised difference VI of reflectivity at 531 nm and 570 nm and has been developed for disease detection^43^,

4 $$\begin{aligned} \text {PRI}=\frac{(R_{531}-R_{570})}{(R_{531}+R_{570})}. \end{aligned}$$

The disease water stress index (DSWI) is the ratio between reflectances at 550 nm and 680 nm^44^,

5 $$\begin{aligned} \text {DSWI}=\frac{R_{550}}{R_{680}}. \end{aligned}$$

And the healthy index (HI)^45^ can be expressed by,

6 $$\begin{aligned} \text {HI}=\frac{(R_{534}-R_{698})}{(R_{534}+R_{698})}-0.5R_{704}. \end{aligned}$$

### Conventional decision fusion techniques

The general idea of classifier/decision fusion can be summarised as merging multiple learners or classifiers to produce the best possible decision so as to enhance the prediction performance over a single classifier. By taking into account the outputs of all classifiers, combinations of multiple classifiers minimise the risk of choosing a weak classifier, stabilise results of random classifiers and increase the robustness of the decisions^46^. Classifier/decision fusion has been an active research topic in machine learning since the late twentieth century and much of the effort has been devoted to combining classifiers for decision making in several pattern recognition applications^47–50^. Typically, in a multiple classifier system, there are two general approaches to obtaining the final decision^46^:

1. Selection: Assuming complementary classifiers, only a single selected classifier contributes to the final decision.
2. Fusion: Assuming competitive classifiers, the integration of all classifiers determines the final decision.

Based on the output information of classifiers, fusion can be divided into three levels^51^:

1. Abstract level: Each classifier only outputs the predicted class label for each input. An abstract level combiner includes weighted or unweighted versions of the majority vote.
2. Rank level: For each input, classifiers rank all labels or classes and produce a list of possible predictions.
3. Measurement level: Instead of class labels, each classifier outputs the probability or confidence score for each class. The measurement level contains the most information among these three levels, making it possible to incorporate with various combiners (e.g. average, maximum, minimum and product), by using either selection or fusion methods.

Various methods in the literature are also concerned with how the final outputs can be combined. Majority vote is the simplest and most used combiner, in which the ensemble of classifiers choose the class that receives the highest number of votes. The fusion scheme for the unweighted majority voting can be described as,

7 $$\begin{aligned} {\hat{y}}=\arg \max _{\theta _{j} \in \{\theta _{1},\theta _{j},\ldots ,\theta _{C}\}}\sum _{i=1}^{L}{\hat{y}}_{i,j}, \end{aligned}$$

where $\{\theta _{1},\theta _{j},\ldots ,\theta _{C}\}$ are the *C* possible classes that an input is to be assigned to, *L* denotes the total number of classifiers, ${\hat{y}}_{i,j}$ is the predicted output of the *i*th classifier for the *j*th class, and ${\hat{y}}$ represents the final decision. In cases where each classifier contributes unequally to the fusion output, a weighted majority vote scheme can be employed by associating a weighting $w_{i}$ for *i*th classifier, and the decision becomes,

8 $$\begin{aligned} {\hat{y}}=\arg \max _{j \in \{\theta _{1},\theta _{j},\ldots ,\theta _{C}\}}\sum _{i=1}^{L}w_{i}{\hat{y}}_{i,j}. \end{aligned}$$

Apart from the majority voting, multiple rules can be applied at the measurement level^49,52^. The maximum, minimum or average rule finds the maximum, minimum or average probability of each class among the classifiers and assigns the input to the class with the maximum score among the maximum, minimum or average scores, respectively. These rules can be expressed as,

9 $$\begin{aligned} {\hat{y}}=  \arg \max _{\theta _{j} \in \{\theta _{1},\theta _{j},\ldots ,\theta _{C}\}}\max _{L}P(\theta _{j}|x_{i}), \end{aligned}$$

10 $$\begin{aligned} {\hat{y}}=  \arg \max _{\theta _{j} \in \{\theta _{1},\theta _{j},\ldots ,\theta _{C}\}}(1-\min _{L}P(\theta _{j}|x_{i})), \end{aligned}$$

11 $$\begin{aligned} {\hat{y}}=  \arg \max _{\theta _{j} \in \{\theta _{1},\theta _{j},\ldots ,\theta _{C}\}}\frac{1}{L}\sum _{i=1}^{L}P(\theta _{j}|x_{i}), \end{aligned}$$

where $P(\theta _{j}|x_{i})$ represents the estimated probability for input *x* that the *i*th classifier output $x_{i}$ belongs to the *j*th class $\theta _{j}$. Similarly, the product rule multiplies the probabilities or confidence scores generated by each classifier and assigns the class label with the maximum score to given input pattern,

12 $$\begin{aligned} {\hat{y}}=\arg \max _{\theta _{j} \in \{\theta _{1},\theta _{j},\ldots ,\theta _{C}\}}\prod _{i=1}^{L}P(\theta _{j}|x_{i}). \end{aligned}$$

### Markov random field texture analysis

As a fundamental image property descriptor, image texture models brightness variations in a local neighbourhood. Furthermore, image texture features are associated with various image properties such as orientation, coarseness and smoothness and quantify the spatial arrangements of pixel intensities in an image or an image region. Texture-based image analysis has been shown to be helpful in various applications such as remote sensing, medical imaging and industrial inspection.

MRFs are generative, flexible and stochastic image texture models, in which contextual dependencies and spatial interrelationships are established among image pixels or other correlated features^53^. Due to the random nature of imaging and noise, pixels are naturally considered as random variables that are conditionally related to neighbouring variables. As undirected probabilistic graph models, MRFs not only specify the conditional dependencies between these random variables, but also interpolate the joint probability distributions with useful potential functions^54^. MRF based texture analysis plays an important role in modern texture modelling and synthesis^55–57^ as well as helps visual interpolation and image understanding^54,58,59^.

A typical Gaussian MRF model is a stationary noncausal two-dimensional autoregressive process that can be expressed by a set of difference equations^53,60^ as

13 $$\begin{aligned} f_{s}=\sum _{s+r\in N_{s}}\beta _{r}f_{s+r}+e_{s}, \end{aligned}$$

where *r* is the relative position with respect to central pixel *s*, and $\{e_{s}\}$ is a stationary Gaussian noise sequence with zero mean and standard deviation $\sigma ^{2}$ characterised by

14 $$\begin{aligned} E(e_{s}e_{s+r})=\left\{ \begin{array}{lll} \sigma ^{2}&{}\;\text {if } r=(0,0) \\ -\sigma ^{2}\beta _{r}&{}\;\text {if } r\ne (0,0)\\ 0&{}\;\text {otherwise} \end{array}\right. , \end{aligned}$$

where $\beta _{r}$ is the model parameter describing the relationship between pixels $f_{s}$ and $f_{s+r}$. All the parameters $\beta _{r}$ in the neighbourhood system $N_{s}$ form the parameter vector ${\varvec{\beta }}$.

In model-based texture methods, model parameters can be used as features for distinguishing textures. Model parameter estimation plays an significant role in analysing image properties and the least squares estimation is commonly used to estimate Gaussian MRF models^60^. The quadratic difference $\Theta$ between the centre pixel and its neighbours can be defined as

15 $$\begin{aligned} \Theta =\sum _{s}\Big (f_{s}-\sum _{s+r\in N_{s}}\beta _{r}f_{s+r}\Big )^{2}. \end{aligned}$$

The least squares problem can be resolved by a close-form solution,

16 $$\begin{aligned} \hat{{\varvec{\beta }}}=(F_{s+r}^{T}F_{s+r})^{-1}F_{s+r}^{T}\Theta , \end{aligned}$$

where $f_{s+r}$ represents the neighbouring pixels of $f_{s}$.

### ProbDecFus: probabilistic decision fusion

Support vector machines (SVMs) are commonly used machine learning algorithms for classification and regression. Given training vectors $\varvec{X}=\{\varvec{x}^{(1)},\varvec{x}^{(2)},\ldots ,\varvec{x}^{(N)}\}=\{\varvec{x}^{(k)}\}_{k=1}^{N}\in {\mathbf {R}}^{M\times N}$ and its corresponding class labels $\varvec{Y}=\{y^{(1)},y^{(2)},\ldots ,y^{(N)}\}=\{y^{(k)}\}_{k=1}^{N}$, the $\nu$-SVM^61^ solves the quadratic optimisation problem

17 $$\begin{aligned} &\min _{\varvec{\omega },b,\varvec{\xi },\rho } \frac{1}{2}\varvec{\omega }^{T}\varvec{\omega }-\nu \rho +\frac{1}{N}\sum _{k=1}^{N}\xi _{k}\\ &\text {s.t.}\;\;y^{(k)}(\varvec{\omega }^{T}\phi (\varvec{x}^{(k)})+b)\geqslant \rho -\xi _{k},\\ &\quad \nu \in (0,1], \; \xi _{k}\geqslant 0,\; \rho >0\\  \end{aligned} $$

where $\varvec{\omega }$ denotes the weight vector, *b* is the learning bias, $\xi$ is a non-zero slack variable, and $\nu$ is the regularisation parameter that controls the trade-off between smaller training errors and larger margins. $\nu \in (0,1]$ represents an upper bound on the fraction of training margin errors as well as a lower bound of the fraction of support vectors^61,62^. Training vectors $\varvec{x}_{i}$ are mapped into a high-dimensional space by function $\phi$ though the kernel trick $K(\varvec{x}^{(i)},\varvec{x}^{(j)})=\phi (\varvec{x}^{(i)})^{T}\phi (\varvec{x}^{(j)})$. A radial basis function (RBF) is a typical kernel function

18 $$\begin{aligned} K\big (\varvec{x}^{(i)},\varvec{x}^{(j)}\big )=\exp \big (-\gamma \Vert \varvec{x}^{(i)}-\varvec{x}^{(j)}\Vert ^{2}\big ),\;\gamma >0, \end{aligned}$$

where $\gamma$ is the kernel parameter. Hence the predicted class labels $\varvec{{\hat{Y}}}=\{{\hat{y}}^{(1)},{\hat{y}}^{(2)},\ldots ,{\hat{y}}^{(N)}\}=\{{\hat{y}}^{(k)}\}_{k=1}^{N}$ can be obtained through the decision function,

19 $$\begin{aligned} {\hat{y}}=\text {sigmoid}\bigg (\sum _{k=1}^{N}\alpha _{k}y^{(k)}K(\varvec{x}^{(k)},\varvec{x})+b\bigg ), \end{aligned}$$

where $\alpha _{k}$ is the Lagrange multiplier.

In addition to predicted class labels, it is also possible to obtain an estimated probability for each class, $P(\theta _{j}|\varvec{x}^{(k)})$, by minimising the negative log likelihood and optimising the quadratic problem^62–64^. In this study, three independent SVM classifiers were constructed based on the spectral reflectances, VIs and MRF spatial features, respectively. The spectral reflectance profiles, $\varvec{x}_\text {0}^{(k)}$, extracted from selected areas of leaves, were averaged within the regions over the entire wavelengths. The empirical VI information, $\varvec{x}_\text {VI}^{(k)}$, refers to the concatenation of six VIs (CI, MCARI, TCARI, PRI, DWSI and HI) calculated on the spectral reflectance. The MRF spatial features, $\varvec{x}_\text {MRF}^{(k)}$, were produced by estimating the texture parameters in each of the selected area. The classifier built on the spectral reflectances was considered as the baseline model, and we proposed a probabilistic decision fusion scheme, ProbDecFus, for integrating spectral and spatial information for further classification. Firstly, we calculated a threshold value $\mu$ based on the classification accuracy of the validation set ($\hbox {acc}_\text {val}$),

20 $$\begin{aligned} \mu =\mu _{1}*\text {acc}_\text {val} +\mu _{2}*(1-\text {acc}_\text {val}) \end{aligned}$$

where,

21 $$\begin{aligned} \mu _{1}=\min _{k \in \{y^{(k)}={\hat{y}}^{(k)}\}}\max _{\theta _{j} \in \{\theta _{1},\theta _{j},\ldots ,\theta _{C}\}}P_\text {val}(\theta _{j}|\varvec{x}^{(k)}_{0}) \end{aligned}$$

22 $$\begin{aligned} \mu _{2}=\max _{k \in \{y^{(k)}\ne {\hat{y}}^{(k)}\}}\max _{\theta _{j} \in \{\theta _{1},\theta _{j},\ldots ,\theta _{C}\}}P_\text {val}(\theta _{j}|\varvec{x}_{0}^{(k)}) \end{aligned}$$

Then according to the probability estimations, the final classification results were generated by weighting and fusing the three classifiers using

23 $$\begin{aligned} w_0^{(k)}= 1-\frac{\min _{\theta _{j} \in \{\theta _{1},\theta _{j},\ldots ,\theta _{C}\}}P(\theta _{j}|\varvec{x}_{0}^{(k)})}{\max _{\theta _{j} \in \{\theta _{1},\theta _{j},\ldots ,\theta _{C}\}}P(\theta _{j}|\varvec{x}_{0}^{(k)})} \end{aligned}$$

24 $$\begin{aligned} w_{i}^{(k)}=\left\{ \begin{array}{ll} 1-\frac{\min _{\theta _{j} \in \{\theta _{1},\theta _{j},\ldots ,\theta _{C}\}}P(\theta _{j}|\varvec{x}_{i}^{(k)})}{\max _{\theta _{j} \in \{\theta _{1},\theta _{j},\ldots ,\theta _{C}\}}P(\theta _{j}|\varvec{x}_{i}^{(k)})} &{} w_{0}^{(k)} \leqslant w_{\mu }\\ 0 &{}w_{0}^{(k)} > w_{\mu } \end{array}\right. \end{aligned}$$

## Supplementary Information

Supplementary Information 1.

Supplementary Information 2.

## Data Availability

The MSI dataset of these three trials (Cassava-TME204-UCBSV) are available at https://doi.org/10.5281/zenodo.4636968.
