# Supplementary material for: Early detection of plant virus infection using multispectral imaging and spatial–spectral machine learning
Source: Sci Rep. 2022 Feb 24;12:3113. doi: 10.1038/s41598-022-06372-8 (PMC8873445; doi:10.1038/s41598-022-06372-8)
Supplement: Supplementary file 1 — Supplementary Information 1. [file 41598_2022_6372_MOESM1_ESM.docx]

Fig. 1(C)

End-point RT-PCR of total RNA extracts isolated from untreated (U), mock-inoculated (M), and UCBSV-inoculated plants at 88 dpi. The upper panel shows a 445-bp band corresponding to UCBSV panel.

The RbcS panel shows a 619-bp band corresponding to a cassava RbcS transcript, which served as a positive control for the isolation of amplifiable RNA.
