# Supplementary figures and images for: Early detection of plant virus infection using multispectral imaging and spatial–spectral machine learning
Source: Sci Rep. 2022 Feb 24;12:3113. doi: 10.1038/s41598-022-06372-8 (PMC8873445; doi:10.1038/s41598-022-06372-8)

Fig. 1C

UCBVS

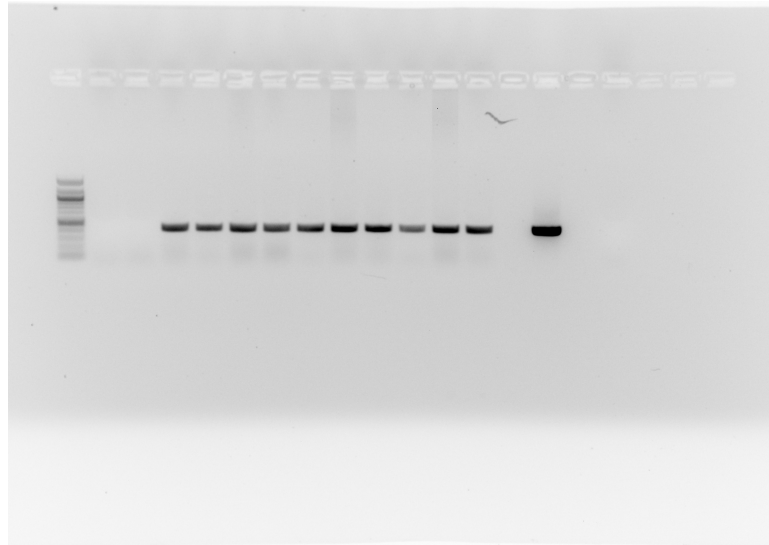

RbcS

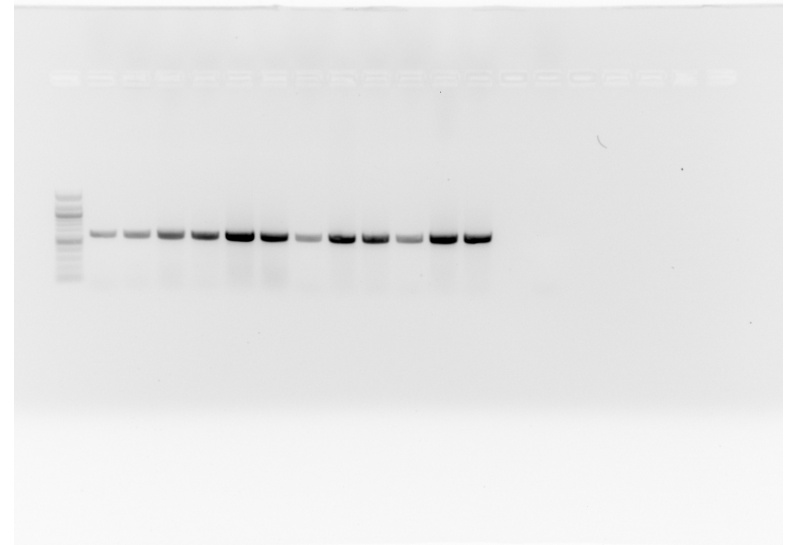

Supplement: Supplementary file 2 — Supplementary Information 2. [file 41598_2022_6372_MOESM2_ESM.pdf]
